# The applicability research of the diagnostic criteria for 10.2 Heamodialysis-related headache in the international classification of headache disorders-3^rd^ edition

**DOI:** 10.1186/s10194-023-01548-7

**Published:** 2023-02-28

**Authors:** Ying Yang, Fanchao Meng, Hanyu Zhu, Lei Zhang, Guangshuang Lu, Shaobo Xiao, Jiaji He, Shengyuan Yu, Ruozhuo Liu

**Affiliations:** 1 Chinese PLA Medical School, Fuxing Road 28, Haidian District, Beijing, 100853 PR China; 2 Department of Neurology, the First Medical Centre of Chinese PLA General Hospital, Fuxing Road 28, Haidian District, Beijing, 100853 PR China; 3 Blood Purification Center, the First Medical Center of Chinese PLA General Hospital, Fuxing Road 28, Haidian District, Beijing, 100853 PR China; 4 The Lu’an Hospital Affiliated to Anhui Medical University, the Lu’an People’s Hospital, Wanxi Road 21, Jinan District, Lu’an, 237000 PR China

**Keywords:** ICHD-3, Diagnostic criteria, Headache, Haemodialysis, Dialysis

## Abstract

**Background:**

Headache during hemodialysis (HDH) is prevalent but not negligible. Despite the high prevalence of dialysis headaches, they have rarely been studied. Therefore, this study aimed to evaluate the prevalence, risk factors, and clinical characteristics of HDH and reappraise the HDH diagnostic criteria in the International Classification of Headache Disorders 3 (ICHD-3).

**Methods:**

One hundred and fifty-four patients completed this randomized cross-sectional study. Consecutive patients who underwent haemodialysis were assessed using a semi-structured questionnaire. The patients were administered face-to-face questionnaires while undergoing dialysis.

**Results:**

This study included 154 patients. Before commencing dialysis, 3.24% (5/154) of the patients had migraine without aura, 1.29% (2/154) had menstrual-related migraine, 0.6% (1/154) had tension-type headaches, and 0.6% (1/154) had an unclassifiable headache. One case (0.6%) of headache resolved after dialysis treatment. HDH was diagnosed in 9.09% (14/154) of the patients. Headache after haemodialysis (HAH) was reported in 6.49% (10/154) of patients. The most prevalent features of HDH were frontal or temporal location, bilateral headaches, dull and throbbing nature, and moderate severity. HDH started at a mean of 2.33 ± 0.79 h after dialysis commenced. The average headache duration was 6.56 ± 1.57 h (median = 3.0 h), with 66.67% of the patients reporting a duration of ≤4 h. HDH was more prevalent in females than males (*P* = 0.01, *P* < 0.05). Female sex was a risk factor for HDH (*P* = 0.01，*P* < 0.05).

**Conclusions:**

The diagnostic criteria for 10.2 HDH in ICHD-3 may miss several HAH. Therefore, ICHD-3 should be revised according to the literature and further studies.

## Introduction

Headache is one of the most common neurological symptoms in patients undergoing dialysis [1]. Previous studies have reported that the prevalence of dialysis headache ranges from 6.6% to 70% [2–5].

There is no consensus regarding the pathophysiology or triggering factors of headache during haemodialysis (HDH). Factors associated with dialysis headaches include sodium ion, magnesium ion [6], urea, blood pressure changes, and weight levels; calcitonin gene-related peptide and substance P levels during dialysis [7]; and different haemodialysis (HD) modalities (standard HD and online hemodiafiltration technique (OL-HDF), standard HD presents a higher risk of dialysis headache than OL-HDF) [8].

Table 1 shows the International Classification of Headache Disorders 3 (ICHD-3) version published in 2018 and the diagnostic criteria for HDH (Chapter 10.2). Patients classified as having dialysis headaches must fulfil the evidence of causation by at least one of the following:

**Table 1:** The international classification of headache disorders, 3rd edition [9]

10.2 The diagnostic criteria of headache during dialysis	
Headache with no specific characteristics occurring during and caused by hemodialysis. It resolves spontaneously within 72 hours after the hemodialysis session has ended.	
A. At least three episodes of acute headache fulfilling criterion C	
B. The patient is undergoing hemodialysis	
C. Evidence of causation demonstrated by at least two of the following:	
1. each headache has developed during a session of hemodialysis	
2. either or both of the following:	
a) each headache has worsened during the dialysis session	
b) each headache has resolved within 72 hours after the end of the dialysis session	
3. headache episodes cease altogether after successful kidney transplantation and termination of hemodialysis	
D. Not better accounted for by another ICHD-3 diagnosis.	
Caffeine is rapidly removed by dialysis: 8.3.1 Caffeine-withdrawal headache should be considered in patients who consume large quantities of caffeine.	

1. the headache must have started during the haemodialysis session, or 2. the headache need to have worsened during the haemodialysis session or resolved within 72 h after the session ended [9]. In our clinical practice, we discovered that some patients’ headaches occurred after dialysis. This type of headache has an obvious causal and time-dependent relationship with dialysis. According to ICHD-3 diagnostic criteria, 10.2. C1, this headache type could not be classified as HDH.

After the occurrence of a headache, neurologist consultation may be required, and further examination may increase the clinical burden. HDH causes patients to experience more pain and makes treatment and nursing more difficult for neurology, nephrology, and pain specialists. Despite the high prevalence of dialysis headaches, they have rarely been studied. Its diagnosis, characteristics, and management remain challenging for neurologists and nephrologists [10].

Therefore, this study aimed to examine the frequency, clinical characteristics, and possible associated factors of HDH and discuss the diagnostic criteria proposed by the International Headache Society.

## Methods

This study was approved by the Research Ethics Committee of the Chinese People’s Liberation Army (PLA) General Hospital (Ethics No. S2022–536-01). All patients who participated in this study signed an informed consent statement.

The study period was from January 2022 to September 2022. Patients with chronic kidney failure who underwent haemodialysis at the First and Second Blood Purification Centre of the First Medical Centre of Chinese PLA General Hospital received dialysis treatment for at least 6 months. In these services, bicarbonate was used in the dialytic solution, and the duration of the haemodialysis sessions was 4 h. All patients regularly underwent three weekly dialysis treatments: Monday, Wednesday, and Friday or Tuesday, Thursday, and Saturday. Patients were enrolled randomly, that is, on random weekdays. (All patients were regularly undergoing three dialysis treatment per week, such as on Monday, Wednesday and Friday or on Tuesday, Thursday and Saturday. We randomly selected some weekday mornings to complete questionnaire by face-to-face interview.)

### Exclusion criteria involved

1. Patients with heart insufficiency, stroke, tumour, uraemic encephalopathy, and other serious diseases;
2. patients with a history of drug abuse, withdrawal, poisoning, trauma, or epilepsy that may be related to headaches;
3. patients who have cognitive, visual, speech, or hearing impairment, or cannot complete the scale assessment and are critically ill, unable to cooperate, or have serious consciousness and mental disorders; and
4. Patients who refused to fill out the questionnaire.

We prepared a semi-structure questionnaire examining the presence and clinical characteristics of headaches before and during the haemodialysis program. The questionnaire consisted of three parts.

- Part 1: This study collected basic information, including sex, age, occupation, whether there was a dialysis headache, whether there was a previous headache, dialysis history, and comorbidities of haemodialysis patients, including hypertension, diabetes, hyperkalaemia, hyperlipidaemia, hyperphosphatemia, hyperuricaemia, secondary hyperparathyroidism, renal anaemia, uraemia, coronary heart disease, and whether there was a cause of secondary headache.
- Part 2: Information on headaches before dialysis were collected, including the occurrence, duration, location, quality, intensity, accompanying symptoms, improvements after rest, worsening of daily activities, and manner of relief.
- Part 3: Information on headaches during dialysis were collected, including dialysis history, how long after dialysis initiation the patient began to experience a headache, how much time after dialysis the patient began to experience a headache, occurrence, duration, location, quality, intensity, accompanying symptoms, improvements after rest, worsening of daily activities, and manner of relief. The intensity was assessed using a numeric rating scale, a segmented numeric version of the visual analogue scale (VAS), where patients select a whole number from 0 to 10 based on their pain intensity [11].

Two headache experts performed extensive face-to-face physical and neurological examinations on all patients. The patients were interviewed face-to-face concerning their headache experiences during the sessions.

The patients were divided into two groups: patients with headaches and control. Patients with headaches were categorized based on their temporal profile relative to dialysis: HDH and headache after haemodialysis (HAH). Patients without headaches were classified as the comparative control group.

### Statistical analysis

Statistical analyses were performed using IBM SPSS Statistics software version 25.0. Continuous data with a normal distribution are reported as means ± standard deviations. Hypotheses on the mean differences between groups were tested using the independent samples t-test or analysis of variance. The median and interquartile range were used to represent non-normally distributed continuous data, and the rank sum test was used for comparison between groups. Categorical data are reported as numbers and percentages. Relationships between categorical variables were analysed using Fisher’s exact or Pearson’s chi-squared test. Multiple logistic regression analyses were performed on variables that were considered clinically significant and with a *p*-value < 0.1 on univariate analysis. Statistical significance was set at *p* < 0.05 using two-tailed testing.

## Results

**Fig. 1:**
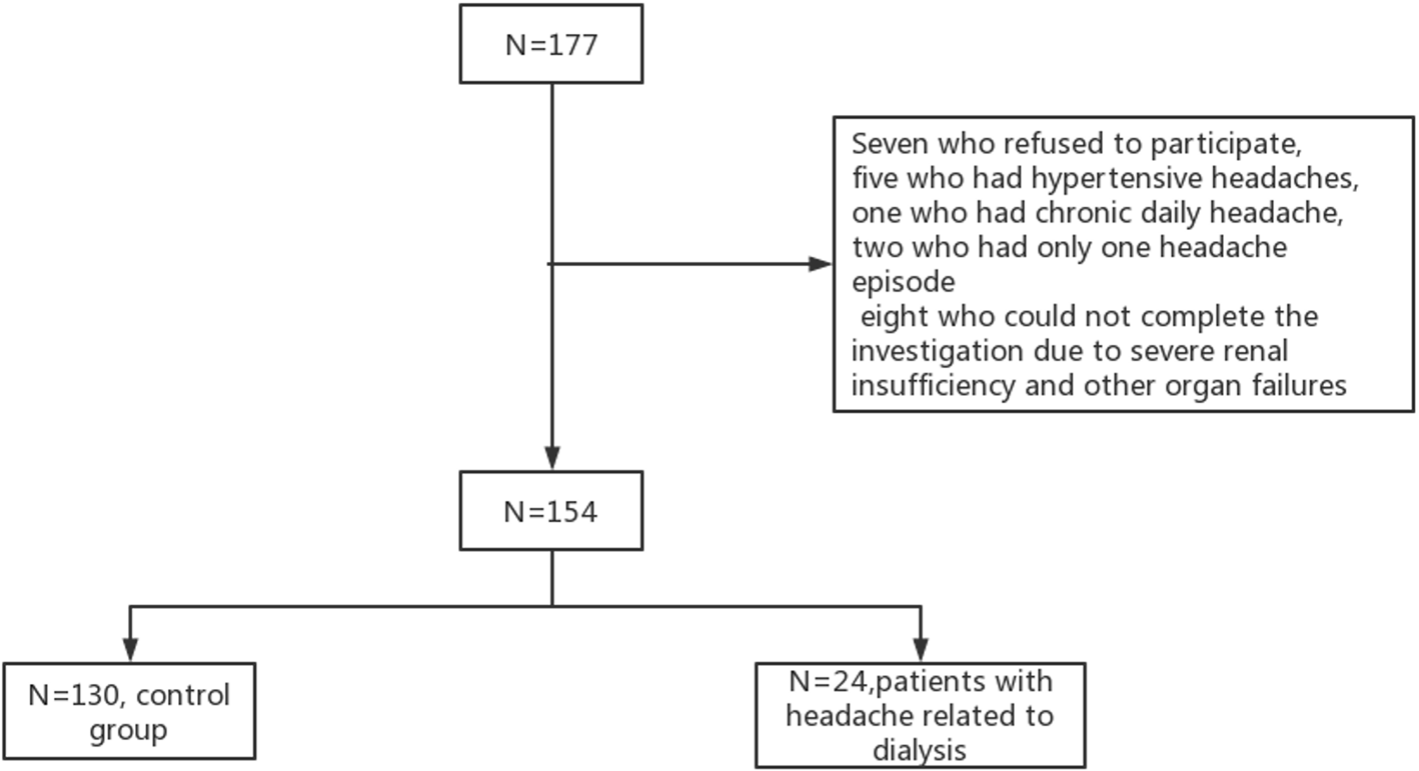
Enrollment of patients

This study enrolled 177 patients. Seven who refused to participate, five who had hypertensive headaches, one who had chronic daily headache, two who had only one headache episode, and eight who could not complete the investigation due to severe renal insufficiency and other organ failures (Fig. 1).

A total of 154 patients completed the study. Of this, 59.09% (91/154) were males, and 40.90% (63/154) were females. Their mean age was (58.54 ± 14.29) years. The mean duration of chronic dialysis therapy was 6.38 ± 5.40 years. Hypertension was present in 94.8% (146/154) of the patients. Diabetes affected 21.4% (33/154), 40.90% (63/154) had coronary heart disease, 25.97% (40/154) had hyperkalaemia, 59.09% (91/154) had hyperlipidaemia, 77.92% (120/154) had hyperphosphatemia, 42.2% (65/154) had hyperuricaemia, 50.64% (78/154) had secondary hyperparathyroidism, 59.09% (91/154) had renal anaemia, and 80.51% (124/154) had uraemia.

Before the dialysis commenced, 5.8% (9/154) of patients had headaches, 3.24% (5/154) had migraine without aura, 1.29% (2/154) had menstrual-related migraine, 0.6% (1/154) had tension-type headaches, and 0.6% (1/154) had an unclassifiable headache. One case (0.6%) of headache resolved after dialysis treatment. Among these patients, six (3.8%) had a dialysis headache with characteristics of some primary headaches.

Twenty-four patients (15.58%) had experienced headaches during or after dialysis therapy (excluding patients with a headache before the beginning of the haemodialysis program). Of this, 25% (6/24) were male, and 75% (18/24) were female, with a mean age of 57.45 ± 10.75 years. The dialysis headache history was 7.64 ± 5.26 years. Of this, 95.8% (23/24) patients with headache had hypertension, 4.2% (1/24) had diabetes, 37.5% (9/24) had coronary heart disease, 16.7% (4/24) had hyperkalaemia, 54.2% (13/24) had hyperlipidaemia, 70.8% (17/24) had hyperphosphatemia, 45.8% (11/24) had hyperuricaemia, 41.7% (10/24) had secondary hyperparathyroidism, 45.8% (11/24) had renal anaemia, and 79.2% (19/24) had uraemia (Table 2).

**Table 2:** Comorbidities of hemodialysis patients

	Headache group (%)	Control group (%)	Total	***P*** values
Hypertension	23(95.8%)	123(94.6%)	146(94.8%)	1
Diabetes	1(4.2%)	32(24.6%)	33(21.4%)	< 0.001
Coronary heart disease (CHD)	9(37.5%)	54(41.5%)	63(40.90%)	0.69
Hyperkalemia	4(16.7%)	36(27.7%)	40(25.97%)	0.25
Hyperlipidemia	13(54.2%)	78(60.0%)	91(59.09%)	0.564
hyperphosphatemia	17(70.8%)	103(79.2%)	120(77.92%)	0.324
Hyperuricemia	11(45.8%)	54(41.5%)	65(42.2%)	0.718
Secondary parathyroidism	10(41.7%)	68(52.3%)	78(50.64%)	0.32
Renal anemia	11(45.8%)	80(61.5%)	91(59.09%)	0.138
Uremia	19(79.2%)	105(80.8%)	124(80.51%)	0.798
Total	24	130	154	

In total, 9.09% (14/154) of the patients had headaches that fulfilled the criteria for dialysis headaches. Of this, 6.4% (10/154) patients complained of headaches after dialysis, and the temporal relationship between headache and dialysis did not satisfy the ICHD-3 diagnostic criteria 10.2 C1.

**Fig. 2:**
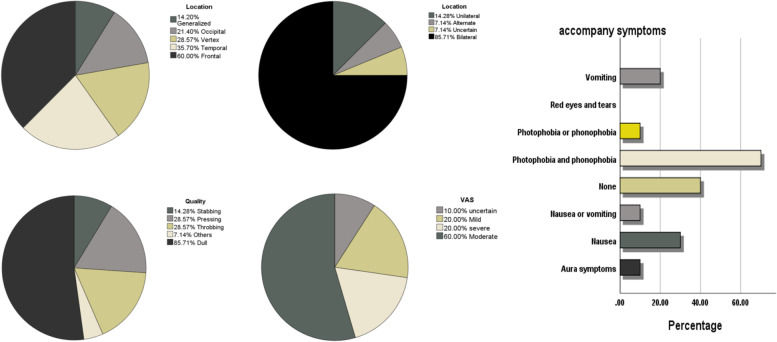
The characteristics of headache during dialysis

Figure 2 illustrates the characteristics of HDH. The HDH group had a mean age of 56.14 ± 10.53 years, and 85.71% were women. The mean duration of haemodialysis was 6.57 ± 5.14 years, while the mean duration of HDH history was 3.96 ± 0.44 years. Headache started at a mean of 2.23 ± 0.79 h after dialysis commenced (median 3.0 h). The mean duration of headache was 6.56 ± 0.47 h, with 66.67% (*n* = 9) of patients reporting duration of ≤4 h. Pain localisation was bifrontal in 60% (*n* = 7), vertex in 28.57% (*n* = 4), bitemporal in 35.7% (*n* = 5), occipital in 21.4% (*n* = 3), and generalised in 14.2% of patients (*n* = 2). Twelve patients (85.71%) had bilateral headaches. The mean VAS score was 5.21 ± 2.0 (median, 5.0). Patients reported experiencing throbbing (4 patients, 28.57%), dull (12 patients, 85.71%), and pressing (4 patients, 28.57%) headaches. This was accompanied by nausea (28.57%, 4/14), vomiting (21.42%, 3/14), nausea or vomiting (14.28%, 2/14), without nausea or vomiting (42.85%, 6/14), and photophobia and phonophobia (78.57%, 11/14). Among them, 64.28% (9/14) were relieved by sleeping and/or massaging. Of the patients, 21.42% (3/14) were relieved without anything, and 14.28% (2/14) were relieved with drugs (Table 4). Furthermore, 55% of patients have less than or equal to 1 seizure per month, 28.57% (4/14) of patients have 2–3 headache episodes per month, and 20% have seizure frequency with each dialysis session.

**Fig. 3:**
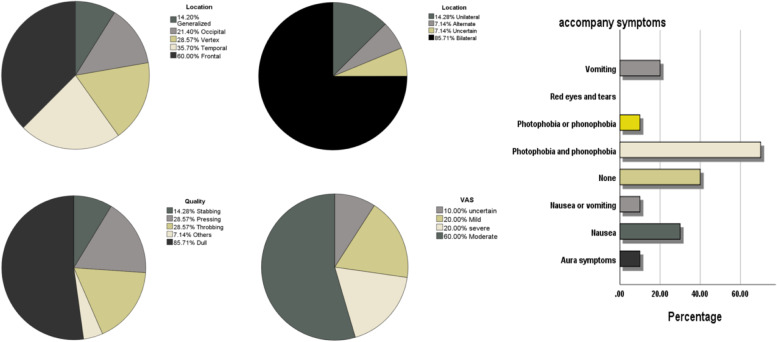
The characteristics of headache after dialysis

Figure 3 depicts the characteristics of HAH. The HAH group had a mean age of 56.8 ± 11.20 years, and 60% were women. The mean duration of haemodialysis was 9.35 ± 4.82 years, and the mean duration of HAH history was 4.92 ± 1.71 years. Headache started at a mean of 1.67 ± 0.50 h after the dialysis ended. The mean duration of headache was 2.24 ± 0.52 h. Pain localisation was bifrontal in 60% (*n* = 6), vertex in 30% (*n* = 3), bitemporal in 40% (*n* = 4), occipital in 10% (*n* = 1), and generalised in 10% (*n* = 1). Six patients (60%) had bilateral headaches. The mean VAS score was 4.78 ± 1.92. Patients reported experiencing throbbing (2 patients, 20%), dull (6 patients, 60%), and pressing (1 patient, 10%) headaches. This was accompanied by nausea (30%, 3/10), without nausea or vomiting (40%, 4/10), and photophobia and phonophobia (70%, 7/10). Among these, 60% (6/10) were relieved by sleep or massage and sleep, 20% (2/10) were relieved without anything, and 10% (1/10) were relieved with drugs.

There were no significant differences in the general characteristics of headache, including headache history (*p* = 0.678), duration (*p* = 0.051), and VAS score (*p* = 0.426) between the HDH and HAH groups (Table 3).

**Table 3:** General characteristics of headache during dialysis and headache after dialysis

Characteristics	HDH	HAH	***P***	Total
History of headache (years)	3.96 ± 0.44	4.92 ± 1.71	0.678	4.32 ± 5.35
Duration (hours)	6.56 ± 1.57	2.24 ± 0.52	0.051	4.94 ± 5.29
VAS score	5.47 ± 2.06	4.78 ± 1.92	0.426	5.21 ± 2
Remission time (hours)	9.76 ± 3.35	4.00 ± 1.46	0.215	7.64 ± 10.84
Headache onset time (hours)	2.23 ± 0.79	1.67 ± 0.50		

*HDH* Headache during hemodialysis, *HAH* Headache after hemodialysis

There were significant differences in sex and diabetes between the HDH or HAH groups and the control group. Patients with primary headaches were inclined to experience dialysis headaches. However, the characteristics of headaches in 80% of patients differed from their previous headaches. No significant differences were observed in age, haemodialysis history, hypertension, hyperkalaemia, hyperlipidaemia, hyperphosphatemia, hyperuricaemia, secondary hyperparathyroidism, renal anaemia, uraemia, or coronary heart disease (Table 2).

## Discussion

In this study, we identified two types of headaches. One is haemodialysis headache, which has been reported using the ICHD-3 diagnostic criteria. Another is HAH, which has a causal relationship with dialysis but does not fulfil the ICHD-3 diagnostic criteria C1. Like Antoniazzi and Jesus [3, 4], we also observed patients who reported having headache episodes between sessions and suspected a relationship with haemodialysis.

### Advise to modify diagnostic criterion ICHD-3 10.2 HDH

#### Criterion C1: each headache developed during a haemodialysis session

In ICHD-3, the criterion C1 is *“each headache developed during a haemodialysis session.”* In our study, 10 patients (6.49%) had headaches after haemodialysis. No significant differences were observed in the characteristics of headache attacks during and after dialysis. HAH had a significant temporal relationship with dialysis. However, 6.49% of the patients did not meet the standard C1 diagnosis time range. According to our results, the C1 criterion included only 14 (9.09%) patients with dialysis-related headaches, and the sensitivity of the C1 criterion could be improved if the time range of the C1 diagnostic criterion was expanded, that is, it occurred during dialysis and within a few hours after dialysis. In our study, the headache occurred at a mean of 1.67 ± 0.50 h after dialysis ended, with a maximum of 2.5 h. Therefore, we suggested that criterion C1 be changed to “*each headache occurred during or two and a half hours after haemodialysis*”. We selected the maximum as an extension to avoid missing diagnoses. The specific extension need to take further large simple size and multicenter studies.

#### Advise to include HDH characteristics

Headache diagnosis is mainly based on its clinical characteristics, such as onset pattern, frequency, duration, location, quality, intensity, accompanying symptoms, and alleviating factors [12, 13]. In ICHD-3, the diagnostic criteria for 10.2 HDH did not describe the clinical characteristics of headache. We summarised the clinical characteristics of HDH in the previous literature and our own research, which are shown in Table 4 [3, 5, 8, 14–17], and discovered that HDH has some common characteristics, such as moderate intensity and bilateral frontal-temporal lobe, with a mean duration of less than 4 h. Therefore, it is suggested to add a diagnostic criteria D: “*Headache has one of the following characteristics:1) moderate intensity, 2) located at bilateral frontal-temporal lobe, 3) the mean duration less than four hours.*” Based on the above discussion, we recommend Table 5 as 10.2 new diagnostic criteria for dialysis headaches.

**Table 4:** The characteristic of HDH in previous and our study

	Headache onset time during HD (h) (mean SD)	Location	Mean duration	Quality	VAS score	Severity	Associated symptoms
Bana [14]		bifrontal		pulsatile		moderate	nausea and vomiting
		Frontotemporal	≤4 h	pulsatile		moderate	
Stojimirovic [5]	the third hour of HD (52%)	Bilateral (81%)	≤4 h(76%)	non-pulsating (67%)	≥8	intensity	without associated symptoms (67%)
Jesus [3]	–	Temporal (27.3)	≤4 h(72.7%)	Throbbing (81.8%)		Moderate (63.6%)	Phonophobia (36.4%)
Göksan [15]	–	fronto-temporal (50%)	< 4 h (63%)	Throbbing (87%)		Moderate (73%)	–
SOUSA MELO [16]	The fourth hour (69.4%)	Bilateral	215.2 (±429.2) minutes	Throbbing (73.5%)	6.7 (±2. 1)	Moderate	Phonophobia (42.9%)
Gozubatik [17]	2.90 ± 0.86 h (median 3.0 h)	Bifrontal (95.4%)	≤4 h (64.0%) (6.22 ± 7.8 h, mean = 3 h)	Throbbing (41.7%) Dull (39.4%)	5.64 ± 2.05	Moderate	Phonophobia (42.3%) Photophobia (40.6%)
Hazim [8]		Bitemporal(62%)	7.4 ± 8.6 h	pulsatile (38%)		moderate (48%)	nausea, vomiting, and photophobia(75%)
Our study	2.23 ± 0.79 h (media 3.0 h)	bilateral (85.71%)frontal(60%)	≤4 h(66.7%)	Dull(85.71%)	5.21 ± 2.0 (median 5.0)	moderate	photophobia and phonophobia (78.57%)

*HD* Hemodialysis, *HDH* Headache during hemodialysis. Moderate: 3–7 in VAS score; Severe: 8–10 in VAS score

**Table 5:** Recommended diagnostic criteria for dialysis-related headache

10.2 The diagnostic criteria of headache during dialysis	
Headache occurring during and caused by hemodialysis. It resolves spontaneously within 72 hours after the hemodialysis session has ended.	
A. At least three episodes of acute headache fulfilling the criteria C	
B. The patient is undergoing hemodialysis	
C. Evidence of causation demonstrated by at least two of the following:	
1. each headache occurred during or two and a half hours after haemodialysis	
2. either or both of the following:	
a) each headache has worsened during the dialysis session	
b) each headache has resolved within 72 hours after the end of the dialysis session	
3. headache episodes cease altogether after successful kidney transplantation and termination of hemodialysis	
D. Headache has one of the following characteristics	
1) moderate intensity	
2) located at bilateral frontal-temporal lobe	
3) the mean duration less than four hours	
E. Not better accounted for by another ICHD-3 diagnosis.	
Caffeine is rapidly removed by dialysis: 8.3.1 Caffeine-withdrawal headache should be considered in patients who consume large quantities of caffeine.	

#### HDH prevalence

Our study revealed that the prevalence rate of HDH was 9.09%. Biljana Stojimirovic et al. [5] evaluated 318 individuals, and 21 (6.6%) had the same complaint. Previous studies reported a 70% prevalence of HDH [14] however, more recent studies reported HDH in 35.4–44% of patients undergoing haemodialysis [16, 17]. The substantial decrease in HDH prevalence may be explained by advances in haemodialysis quality[5] [5]. We excluded patients who has headache prior to dialysis, which may cause the decrease of HDH prevalence.

#### HDH was more often present in female patients

In our study, HDH was more prevalent in females, consistent with the findings of Goksan et al. [15] and Sousa Melo et al. [16]. Other studies have reported that HDH is more prevalent in males [4, 5, 17]. The sex difference may be due to different studies using different methods, which may have enrolled more male patients undergoing haemodialysis. Another possible explanation is that women have lower pain thresholds and fluctuations in sex hormones, especially oestrogen and progesterone, may cause a change in the prevalence or intensity of headaches.

#### Dialysis may be a trigger factor

Jesus et al. [3] reported that 60.1% of patients with a headache history complained of HDH, indicating that these patients were more susceptible to headaches. In our study, 5.8% (9/154) of patients had headaches before commencing dialysis; among these patients, 66.67% (6/9) also complained of HDH. Dialysis may trigger a primary headache attack among patients who have headaches after dialysis and a primary headache.

#### The possible risk factors of headaches related to dialysis

Davidovits demonstrated that headache was associated with haemodialysis, the number of medications used by patients, high phosphor levels, chronic kidney disease grade, lower glomerular filtration rate, anaemia ferritin, anaemia, and a higher parathyroid hormone level [18]. However, in our study, we compared the possible risk factors, including age, dialysis history, and comorbidities in patients undergoing haemodialysis, including hypertension, diabetes, hyperkalaemia, hyperlipidaemia, hyperphosphatemia, hyperuricaemia, secondary hyperparathyroidism, renal anaemia, uraemia, and coronary heart disease between HDH and haemodialysis patients, and observed no significant differences. This may be explained by differences in the study method, dialysis scheme, and race.

### Strengths and limitations

This study discovered that some dialysis headaches occurred after dialysis ended, which may compensate for the ICHD-3 diagnostic criteria of 10.2 dialysis-related headaches. However, our study had some limitations. We did not follow up with these patients for an extended period. Finally, we did not re-examine these symptoms in a prospective study. Further longitudinal follow-up may improve data reliability.

## Conclusion

Haemodialysis-related headache is prevalent in clinical practice but has rarely received attention. The prevalence of dialysis headaches in our study was 15.58% (24/154). The risk factors in patients with headaches were female sex and diabetes, and dialysis may trigger a primary headache episode among patients who had headaches after dialysis and a primary headache.

We discovered that some patients whose headaches did not occur until after dialysis might be omitted. Therefore, we propose some recommendations to improve the practicability of the diagnostic criteria.

## Data Availability

The datasets used and analyzed during the present study are available from the corresponding authors upon reasonable request.

## Abbreviations

HDH: Headache during haemodialysis
HAH: Headache after haemodialysis
HD: Haemodialysis
ICHD-3: International Classification of Headache Disorders, 3rd edition
PLA: People’s Liberation Army
VAS: Visual analogue scale
